# *Drosophila* Araucan and Caupolican Integrate Intrinsic and Signalling Inputs for the Acquisition by Muscle Progenitors of the Lateral Transverse Fate

**DOI:** 10.1371/journal.pgen.1002186

**Published:** 2011-07-21

**Authors:** Marta Carrasco-Rando, Antonio S. Tutor, Silvia Prieto-Sánchez, Esther González-Pérez, Natalia Barrios, Annalisa Letizia, Paloma Martín, Sonsoles Campuzano, Mar Ruiz-Gómez

**Affiliations:** 1 Centro de Biología Molecular Severo Ochoa, Consejo Superior de Investigaciones Científicas–Universidad Autónoma de Madrid, Madrid, Spain; 2 Institut de Biologia Molecular de Barcelona, Consejo Superior de Investigaciones Científicas, Barcelona, Spain; Harvard Medical School and Howard Hughes Medical Institute, United States of America

## Abstract

A central issue of myogenesis is the acquisition of identity by individual muscles. In *Drosophila*, at the time muscle progenitors are singled out, they already express unique combinations of muscle identity genes. This muscle code results from the integration of positional and temporal signalling inputs. Here we identify, by means of loss-of-function and ectopic expression approaches, the Iroquois Complex homeobox genes *araucan* and *caupolican* as novel muscle identity genes that confer lateral transverse muscle identity. The acquisition of this fate requires that Araucan/Caupolican repress other muscle identity genes such as *slouch* and *vestigial*. In addition, we show that Caupolican-dependent *slouch* expression depends on the activation state of the Ras/Mitogen Activated Protein Kinase cascade. This provides a comprehensive insight into the way Iroquois genes integrate in muscle progenitors, signalling inputs that modulate gene expression and protein activity.

## Author Summary

In *Drosophila*, as in vertebrates, the muscular system consists of different types of muscles that must act in coordination with the nervous system to control the adequate release of contraction power required for the proper functioning of the organism. Therefore, the acquisition of specific identities by individual muscles is a key step in the generation of the muscular system. In *Drosophila*, muscle progenitors (specific myoblasts that seed the formation of mature muscles) integrate positional and temporal signalling inputs, resulting in the expression of unique combinations of muscle identity genes, which confer on them specific fates. Up to now, very little was known of how this integration takes place at a molecular level and how a particular code is translated into a specific muscle fate. Here we show that the acquisition of the lateral transverse muscle fate requires the repression mediated by Araucan and Caupolican, two homeoproteins of the Iroquois Complex, of other muscle identity genes, like *slouch* and *vestigial*. The repressor or activator function of the Iroquois proteins depends on the activity of the Ras signalling pathway. Therefore, our work places Iroquois genes at a nodal point that integrates signalling inputs and regulates protein activity and cell fate determination.

## Introduction

**Figure 1:**
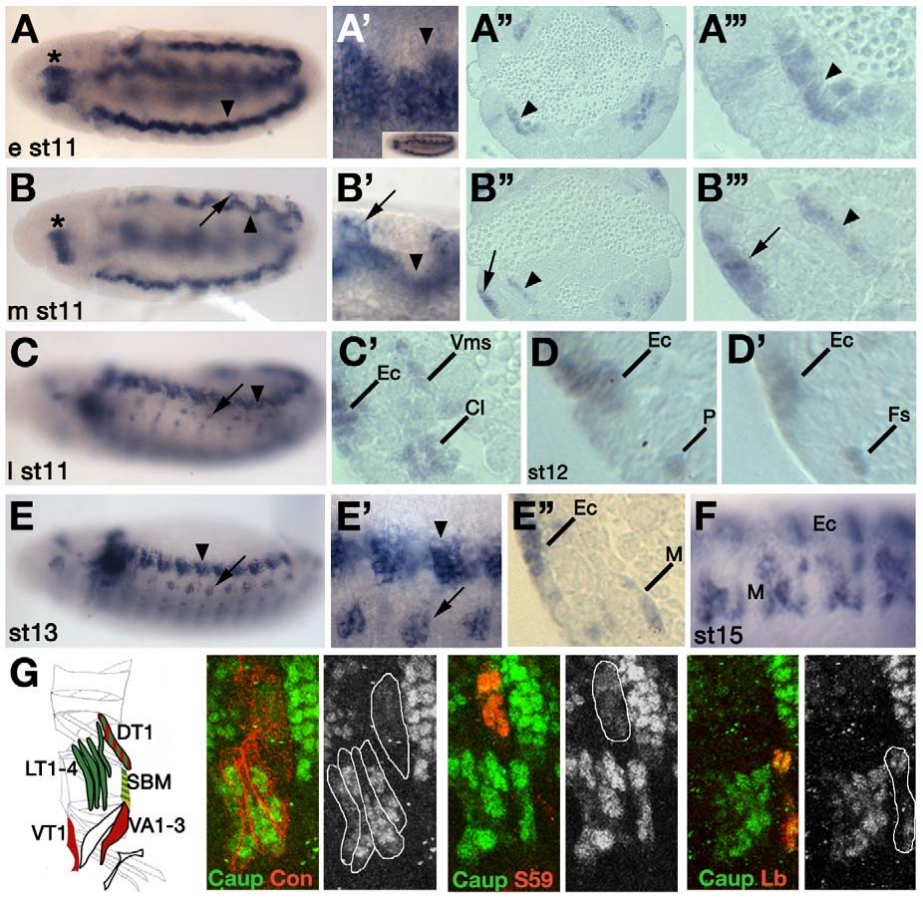
Pattern of expression of *ara* and *caup* during myogenesis. Wild type embryos of the indicated developmental stages were hybridized with *caup* (A, A′, B, B′, C, E, E′) or *ara* (F) riboprobes or sectioned after anti-Caup antibody staining (A″, A′″, B″, B′″, C′, D, D′, E″). (A-A′″) *caup* is expressed in the visceral mesoderm at early stage 11 (arrowheads, A and A′ show the same embryo with different focus as shown in the inset). (B-B′″) At mid stage 11 *caup* is expressed in the visceral mesoderm (arrowheads) and in the lateral ectoderm (arrows). Asterisks in A and B point to the primordium of the proventriculus. A′″, B′″ close-ups of the images shown in A″ and B″, respectively. (C-C′) Early stage 12/late stage 11 embryos. (C) *caup* is expressed in the lateral ectoderm (arrowhead) and in groups of mesodermal cells (arrow). (C′) Cross-section showing *caup* expression in ectodermal cells (Ec), visceral mesoderm (Vms) and promuscular clusters (Cl). (D, D′) At stage 12 *caup* is expressed in individual muscle progenitors (P in D) and slightly later in both founders (Fs) derived from the division of progenitors (D′). (E-E″) At stage 13 Caup is detected in a lateral stripe of ectodermal cells (arrowheads in E, E′, Ec in E″) and in muscle precursors (arrows in E, E′, M in E″). (F) Stage 15 embryo showing expression of *ara* in the ectoderm and in mature muscles. (G) Stage 15 embryos doubled stained with anti-Caup (green) and antibodies against Con, Slou or Ladybird (red). *caup* is co-expressed with *Con* in LT1–4 muscles, with *slou* in DT1 and with *lb* in SBM. The drawing scheme summarises the wild type patterns of expression of *caup* (green), *slou* (red), *lb* (yellow) and *Con* (black contour line) in relation to the wild type complement of abdominal muscles. For muscle nomenclature see [1].

In *Drosophila* as in vertebrates the proper function of the muscular system relies on the generation of a stereotyped pattern of discrete muscles and their intimate connection with the nervous system, which together control the adequate release of contraction power to fulfil the functional requirements of the organism. The formation of a muscle pattern is therefore of great importance and consequently many efforts have been devoted to solve the central problem of the acquisition of muscle identity. The embryonic *Drosophila* muscle pattern comprises thirty elements in each abdominal hemisegment (Figure 1G). Each muscle is a syncytial fibre whose unique characteristics, i.e., position, size, attachment to tendon cells, innervation and pattern of gene expression allow its unambiguous identification [1], [2]. Muscle specification is a stepwise process that ensures the local singling out of a population of myoblasts, the founder myoblasts, each of them containing the necessary information to give rise to a unique muscle. The origin of founder myoblasts can be traced to late embryonic stage 10 when groups of mesodermal cells (the promuscular clusters) start expressing the proneural gene *lethal of scute* and acquire myogenic competence [3]. Opposing activities of Notch and Receptor Tyrosine Kinase signalling pathways ensure that only one cell in the cluster will segregate as a muscle progenitor [4]. This will divide asymmetrically to generate two sibling founder myoblasts or a founder myoblast and an adult muscle precursor [3], [5], [6]. The unselected cells of the promuscular clusters, by activation of the Notch signalling pathway, will initiate the expression of the transcriptional regulator Myoblasts incompetent (also called Gleeful and Lame duck) and become fusion competent myoblasts that by fusing to founders will give rise to multinucleated fibres [7]–[9]. Regarding muscle identity, each progenitor and founder exhibits a specific code of gene expression that confers to muscles their unique characteristics. The components of these codes are accordingly named muscle identity genes (reviewed in [2], [10], [11]). The identity code is transmitted to all the nuclei in the syncytium through the process of myoblast fusion [12]. According to their patterns of expression muscle identity genes can be grouped into three categories. Type I includes genes expressed by progenitors and whose expression is maintained in sibling founders and muscles. Examples are *apterous*, *ladybird* (*lb*) and *Pox meso* (*Poxm*) [13]–[15]. Type II identity genes are expressed in progenitors but differentially regulated in sibling founders, being lost from one of them and the corresponding muscles. Examples are *Krüppel* (*Kr*), *even-skipped* (*eve*), *collier* and *slouch* (*slou*) [3], [4], [16]–[18]. And finally type III refers to genes expressed by progenitors and founders of muscles sharing common characteristics. *vestigial* (*vg*), expressed by all internal muscles, is the only known member of this class [12], [19]. Regarding the onset of their expression a few muscle identity genes, such as *Kr*, *eve* and *collier*, are already expressed in the promuscular cluster, before the segregation of muscle progenitors [4], [16], [18], [20] whereas other genes, like *Connectin* (*Con*), initiate their expression in already segregated progenitors [4], [16], [18], [20].

In this study we identify *araucan* (*ara*) and *caupolican* (*caup*), two members of the Iroquois gene complex (Iro-C), as novel type III muscle identity genes. The Iro-C genes encode homeoproteins conserved throughout the animal kingdom. They are organized in genomic clusters of three paralogous genes, one in the case of *Drosophila* and usually two in most vertebrates [21]. They participate in a wide variety of developmental processes, mainly related to the specification and patterning of diverse territories of the body, including the lateral mesonotum and dorsal cephalic region of *Drosophila*, the neural ectoderm of *Xenopus* and cranial placode derivatives of zebrafish [22]–[30]. Here we show by means of genetic approaches that *ara* and *caup* function redundantly in the specification of the lateral transverse (LT) muscles, since in the absence of both genes LT1–4 muscles loose their LT fates and acquire those of other muscles.

At present there is compelling evidence that muscle progenitors can integrate positional and temporal signalling inputs. This promotes the expression of unique combinations of muscle identity genes, which confers on them their ultimate fate [14]–[18], [31], [32]. There has been extensive analysis on the regulation of some of these genes, such as *eve* and *collier* [4], [33], [34], which has allowed to propose candidate cis-regulatory modules for founder muscle specific expression [35]. However, very little is known about how progenitors integrate the activity of the transcription factors encoded by these genes, about the identity of their direct targets (save in the cases of Kr and Lb [36]–[38]), and of their hierarchical relationships and their putative post-transcriptional regulation. In this report we have focused on these issues in relation to the function of the *ara/caup* identity genes. We demonstrate that the implementation of the lateral transverse muscle fate requires the repression mediated by Ara/Caup of the muscle identity genes *slou* and *vg*, to avoid reiteration of other muscle fates regulated by these transcription factors. In addition, we identify *slou* as a potential direct target of Ara/Caup. Furthermore, our tissue culture and in vivo experiments show that the repression of *slou* by Ara/Caup in LT precursors requires the activity of the Ras/Mitogen Activated Protein Kinase (Ras/MAPK) pathway to be kept at a very low level, since otherwise Caup is converted from a repressor to an activator of *slou*. This is to our knowledge the first evidence of the interplay between the Receptor Tyrosine Kinase signalling pathways and the activity of a muscle identity transcription factor. Therefore, during *Drosophila* embryogenesis, and for the acquisition of the lateral transverse muscle fate, the homeoproteins Ara and Caup appear to act at a nodal point in muscle progenitors, as they integrate positional and temporal signalling inputs that modulate their activity on subordinate identity genes.

## Results

### Expression of Iro-C genes during muscle development

The patterns of expression of *ara* and *caup* in the embryonic ectoderm have been previously reported [39], [40]. In this work we focus on the embryonic *ara* and *caup* mesodermal expression. In situ hybridization showed that here both genes were similarly expressed (Figure 1 and results not shown). At early stage 11 *caup* (and *ara*) transcripts and proteins are detected in groups of cells of the presumptive visceral trunk mesoderm (Figure 1A-1A′″, the available anti-Caup antibody recognises both Ara and Caup proteins). By mid stage 11 they are expressed at the same dorso-ventral level in the visceral mesoderm and in the dorsolateral ectoderm (Figure 1B-1B′″). Expression in the visceral mesoderm declined at late stage 11 when it became detectable in groups of cells of the somatic mesoderm (promuscular clusters [3], Figure 1C and 1C′), from where a subset of muscle progenitors (P) still expressing *ara/caup*, will segregate slightly later (stage 12, Figure 1D). Expression was maintained in sibling founder myoblasts (Fs in Figure 1D′) derived from *ara-caup*-expressing progenitors and in the muscles they give rise to (Figure 1E-1E″), namely LT1–4, dorsal transverse 1 (DT1) and segment border muscle (SBM) (Figure 1F and 1G). The expression in the somatic mesoderm of the third member of the Iroquois complex, *mirror* [41] did not overlap with that of *ara-caup* (not shown).

**Figure 2:**
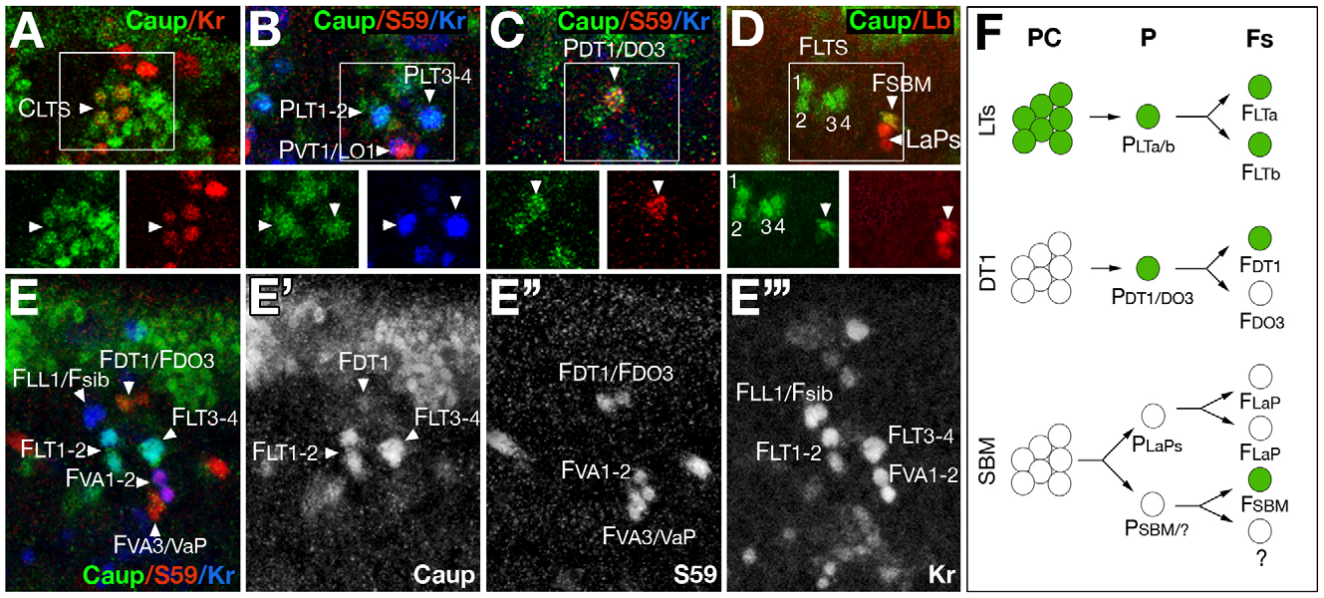
Onset of Caup expression in muscles in relation to other muscle identity genes. All images show a detail of an embryonic wild-type abdominal hemisegment stained with antibodies against Caup (green) and different muscle identity proteins. Images show a ventral view of the embryo, with the exception of B and C that correspond to lateral views. (A–C) Stage 11 embryos. (A) *caup* and *Kr* (red) are co-expressed in a lateral transverse promuscular cluster (C_LTS_). (B–C) *caup* is co-expressed with *Kr* (blue) in progenitors of LT muscles (P_LT1/LT2_ and P_LT3/LT4_, B) and with *slou/*S59 (red) in DT1/DO3 progenitor (P_DT1/DO3_, C). (D) Late stage 12 embryo co-expressing *caup* and *lb* in the SBM founder (F_SBM_). (E) Stage 12 embryo showing co-expression of *caup* with *slou/*S59 (red) in DT1 founder (F_DT1_) and with Kr (blue) in LTs founders (F_LT1–4_). The position of LL1, LL1sib and VA1–3 founders (F_LL1_, F_LL1sib_, F_VA1–3_) and the ventral adult muscle precursor are also indicated. (F) Schematic representation of *ara/caup* expression in the LTs, DT1 and SBM lineages (SBM lineage as revised in [17]). LaPs, lateral adult muscle precursors; PC, promuscular cluster; P, muscle progenitor; Fs, founder myoblasts.

The early expression of *ara/caup* in all lateral muscles with vertical orientation, suggested a possible role as muscle identity genes. Therefore, we compared their expression with that of several muscle identity genes. For the LT1–4 muscles, *ara/caup* were co-expressed with *Kr* in the promuscular clusters from which progenitors P_LT1/LT2_ and P_LT3/LT4_ are singled out (Figure 2A). *ara/caup* expression was maintained at high levels in both progenitors that also express *Kr* (Figure 2B). Whereas *Kr* expression decayed in founders LT1 and LT3 before the onset of myoblast fusion and in LT2 and LT4 muscles from stage 15 onwards [18], expression of *ara*/*caup* was maintained in the four founders (Figure 2D and 2E). These also expressed *Con*, co-expression that was maintained in the mature LT1–4 muscles (Figure 1G). In the case of muscle DT1, the onset of *ara/caup* expression coincided with that of *Con* and *slou* in the progenitor of DT1 and dorsal oblique 3 (DO3) muscles (Figure 2C) and it appeared to be maintained in DT1 founder (Figure 2E) and mature muscle at low levels (Figure 1G). Finally, *ara/caup* co-expressed with *lb* in the SBM founder (Figure 2D), but were not be detected in the *lb*-expressing progenitor and promuscular cluster. In summary, different muscle lineages expressed *ara/caup* at different steps of the myogenic programme (Figure 2F). In the LT1–4 case *ara/caup* and *Kr* were detected at the earliest lineage stage, that is in promuscular clusters, preceding *Con* expression in progenitors (not shown); in the DT1/DO3 lineage *ara/caup* and *slou* were first detected in the already singled out DT1/DO3 progenitor and in the SBM *ara/caup* expression was first detected in the SBM founder after *lb* expression.

### Ara and Caup are required for specification of lateral transverse muscles

**Figure 3:**
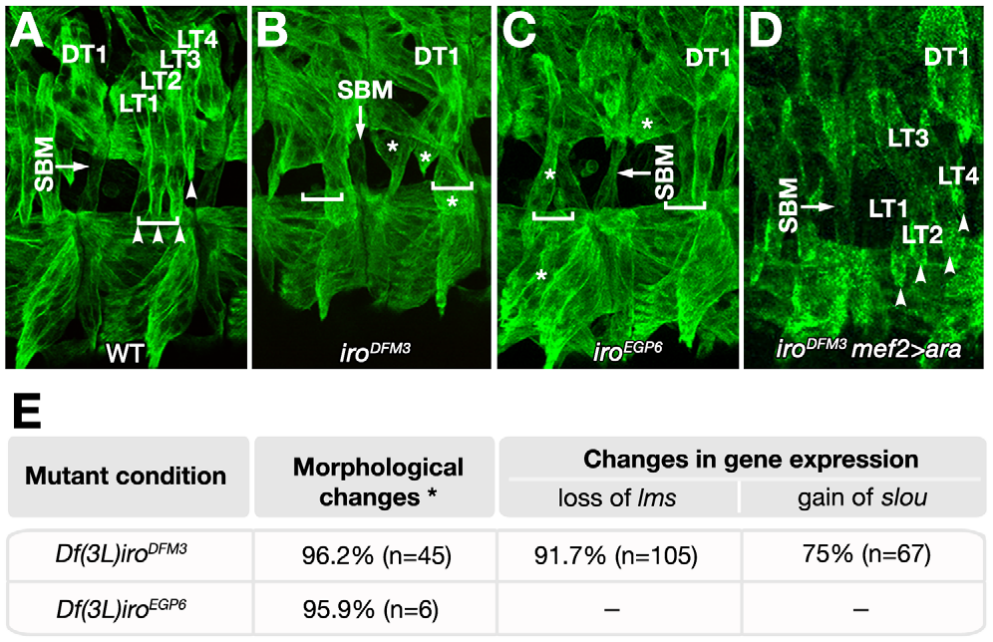
Muscle phenotypes of *Iro-C* mutant embryos. (A–D) Latero-ventral region of stage 16 wild-type (A), *Df(3L)iro^DFM3^* (B), *Df(3L)iro^EGP6^* (C) and stage 15 *Df(3L)iro^DFM3^ mef-2GAL4::UAS-ara* (D) embryos stained with anti- Tropomyosin antibody (green). The position of ventral wild-type LT muscle tips and LT attachment sites are marked with arrowheads and brackets, respectively. Note the absence of muscles with LT morphology and insertions at LT attachment sites, and the presence of morphologically normal DT1 and SBM muscles (arrows) in the mutant backgrounds (B, C). Asterisks indicate morphological abnormal latero-ventral muscles in these embryos. This phenotype is rescued by mesodermal *ara* expression with the pan-mesodermal driver *mef2-GAL4* (D). (E) Quantification of phenotypes produced by the loss of *ara/caup* in LT muscles. * Refers to changes in shape, orientation or attachment sites; n, numbers of hemisegments analysed (stages 14–16); -, not determined.

During imaginal development Ara and Caup can functionally substitute each other in all territories where their function has been investigated [22], [23], [28]. Thus, to analyse their role in embryonic myogenesis and evaluate the possible contribution of *mirror* to any phenotype we might find, we used three deficiencies: *Df(3L)iro^DFM3^*, which removes both *ara* and *caup*, (and probably affects *mirror* regulation, [23], [28]), *Df(3L)iro^EGP6^*, which removes *ara* and *caup* without affecting *mirror* and its regulatory region, and *Df(3L)iro^EGP5^*, which only removes *mirror* [42]. Whereas *Df(3L)iro^EGP5^* embryos did not show any detectable phenotype in the lateral region (not shown), a distortion of the lateral larval muscle pattern (visualised with antibody MAC141 to Tropomyosin) was found in both *Df(3L)iro^DFM3^* and *Df(3L)iro^EGP6^* embryos (Figure 3A–3C). In more than 95% of cases muscles with LT morphology were absent (Figure 3E). Instead, some fibres with abnormal orientation appeared in the lateral and ventral regions, but never inserted at the LT attachment sites (asterisks in Figure 3B and 3C). The loss of LT muscles was further verified by loss of expression of the specific LT muscle marker *CG13424*, recently renamed *lateral muscles scarcer* (*lms*) [43] at stage 15 and the absence of *Con* expression in the lateral somatic mesoderm (Figure S1). Both DT1 and SBM fibres developed with normal morphologies (Figure 3A–3C and Figure S1). To examine the individual contribution of *ara* and *caup* to the phenotype we resorted to embryos mutant for only one of these genes (*ara* in *ara^rF209^*, [28], or *caup* in *iro^EGPΔ1^*, [42]). The larval muscle pattern was normal in both mutants (not shown). Thus, similarly to imaginal development, *ara* and *caup* appear to play redundant roles during embryonic myogenesis.

The absence of muscles with LT morphology in *ara/caup* mutants could be due to a failure of otherwise well specified muscles to find the right insertion to tendon cells, due to ectodermal requirement of Iro-C genes, or to a misspecification of the muscles. Two independent results indicated that Iro-C genes are required autonomously in the mesoderm to specify the LT fate. First, the normal development of LT muscles in *Df(2L)5* embryos devoid of Iro-C gene expression at the ectoderm (Figure S2 and [44]). And second, the rescue of the muscle phenotype of *Df(3L)iro^DFM3^* embryos by Ara supplied exclusively in the mesoderm (using myocyte enhancer factor 2 (*mef2*)*-GAL4* as driver, Figure 3D).

### All progenitors and founders segregate in *Df(3L)iro^EGP6^* mutant embryos

**Figure 4:**
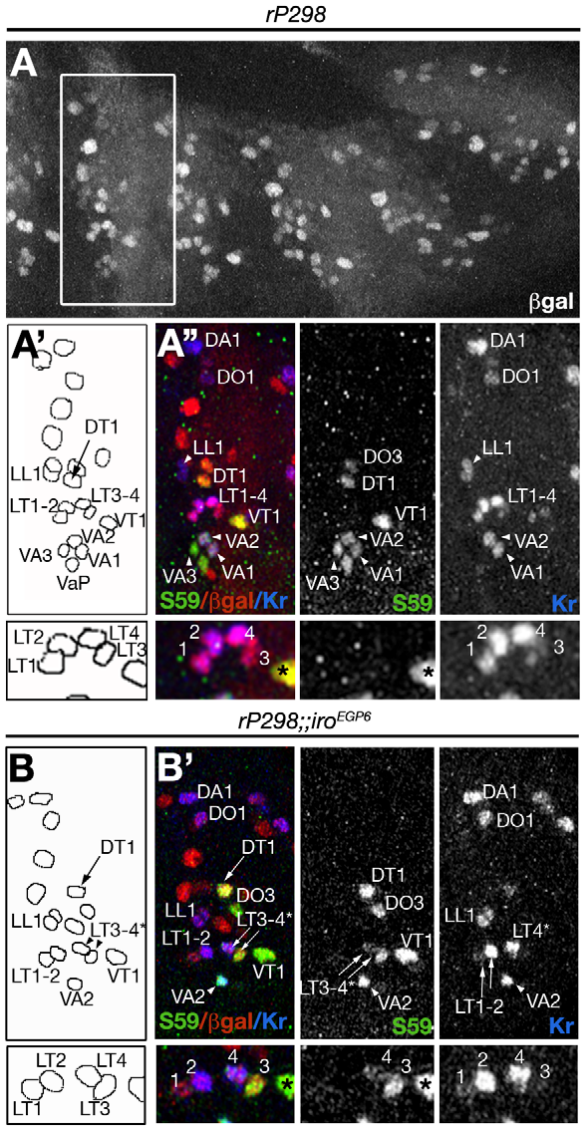
Changes of fate in LT founders of *Iro-C* mutant embryos. (A-B′) Late stage 12 control (A-A″) and *rP298;;Df(3L)iro^EGP6^* sibling embryos (B, B′) stained with anti-ßgal (red), anti-Slou/S59 (green) and anti-Kr (blue) antibodies. ßgal staining is used as a marker for founders (*rP298* line) and the white rectangle in A marks the individual segment shown in A′, A″. (A′, B) Drawings indicating the relative position of the founders visualised in the corresponding (A″, B′) confocal images. The founders expressing *Kr* or *slou*/S59 are labelled by their muscle's acronyms. Note that although founder segregation is unaffected in *Df(3L)iro^EGP6^* embryos, the specification of LT founders is altered (B, B′). Thus, two of the LT founders (LT3–4* in B, B′), marked by expression of *Kr*, also express *slou*/S59, a property exclusive of the VA1–2 founders (see insets below for details of LT founders, the asterisks mark VT1 founder, that expresses *slou*/S59 but not *Kr*. Note that Kr is disappearing from LT1 and 3). All panels show Z projections of several consecutive confocal sections with the exception of A″ that corresponds to a combination of two Z projections, one lateral, as the one shown in B′, and other rotated ventrally to show VAs founders. For muscle nomenclature other than ventral adult precursor (VaP) see [1].

**Figure 5:**
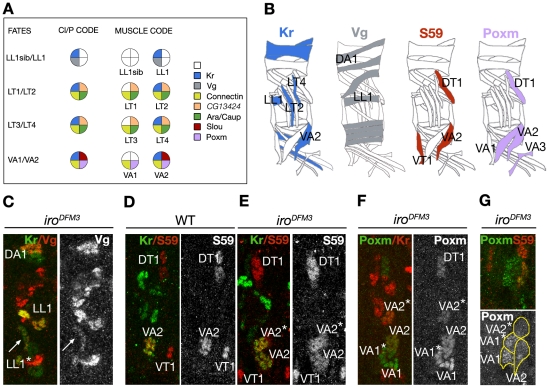
Muscle fate transformations in *Df(3L)iro^DFM3^* embryos. (A) Summary of identity codes for promuscular clusters (Cl), progenitors (P) and muscles missing or duplicated in *ara/caup* mutants, indicated by a colour code. (B) Schematic drawings of the body wall muscles in wild type abdominal hemisegments, depicting the muscles that express the marker indicated on top. (C) Stage 14 *Df(3L)iro^DFM3^* embryo showing a duplication of LL1 fate in the lateral region, pointed by an arrow (LL1*). As shown in the corresponding schemes, LL1 is the only muscle that co-expresses *Kr* (green) and *vg* (red) in the lateral region. (D, E) Double-staining with anti-Kr (green) and anti-Slou/S59 (red) antibodies in stage 14 wild-type (D) and *Df(3L)iro^DFM3^* (E) embryos, showing duplication of VA2 fate in the mutant embryo that co-expresses *Kr* and *slou/*S59 (VA2*). (F) At stage 14 two VA2-like muscle precursors expressing *Kr* and *Poxm* and two *Poxm*-expressing VA1-like precursors are observed in *Df(3L)iro^DFM3^* embryos. (G) The duplicated muscles are clearly visualised at stage 15, when *Poxm* expression is still clear in VA1 but fading in VA2 muscles. Note the presence of two muscles expressing higher levels of *Poxm* (green, VA1 and VA1*) next to two fibres co-expressing low levels of *Poxm* and *slou/*S59 (red) in a *Df(3L)iro^DFM3^* embryo.

We next examined whether the loss of LT muscles was due to either a failure in the segregation of muscle progenitors (absences and/or duplications) or to an early transformation of the fate of LT progenitors. To discern between these possibilities we combined the reporter line *rP298*, which expresses ß-galactosidase in all progenitors and founders [32], [45] with *Df(3L)iro^EGP6^*. We focussed on the previously well-established muscle lineages labelled by Slou/S59 [3], [17] and the LT1–4 lineages labelled by Kr [18]. With these markers in the lateral-ventral region of *rP298* embryos we can identify the following founders (Figure 4A-4A″ and insets below). In the dorsalmost lateral mesoderm we find the sibling founders DT1 and DO3 (expressing *slou*) and the lateral longitudinal 1 (LL1) founder and its sibling (expressing *Kr*). Immediately below segregate the four LT founders (expressing *Kr*). And more ventrally appear the sibling ventral acute 1 (VA1) and VA2 founders (which express *Kr* and *slou*) and the VA3 founder and its sibling, the ventral adult precursor (that express *slou*). In *Df(3L)iro^EGP6^* embryos we observed the same number of identifiable founders (Figure 4B and 4B′). There were however significant differences in terms of patterns of gene expression. Namely, the presumptive LT3–4 founders now expressed *slou* in addition to *Kr* (Figure 4B and 4B′ and insets below). This code of muscle identity gene expression is similar to that of VA1 and VA2 founders (Figure 5A), suggesting an early transformation of LT3–4 to VA1–2 muscles.

### Ara and Caup implement LT muscle fate by repression of muscle identity genes in progenitors

The absence of all muscles with LT morphology in *ara/caup* mutant embryos prompted us to examine whether, in addition to the putative transformation of LT3–4 towards VA1–2, there was a similar change of fate for LT1–2. LT progenitors express *Kr*, *caup*, *Con* and *lms*, P_LL1/LL1sib_ expresses *Kr* and *vg*, and P_VA1/2_ *Kr*, *slou*, *Con* and *Poxm* (Figure 5A and 5B). Using a combination of these markers we found in the lateral region of *Df(3L)iro^DFM3^* embryos an ectopic muscle that expressed Kr+Vg, the code of LL1 (LL1*, Figure 5C) and an ectopic muscle VA2 (VA2* in Figure 5E–5G). This change of muscle identity could take place in founders or at the progenitor state. If this were the case, we anticipated that both muscles resulting from sibling founder myoblasts should be duplicated in *Df(3L)iro^DFM3^* embryos. Indeed, using anti-Poxm, which labels VA1–3 ([14] and Figure 5B), and antibodies to Kr and Slou, which are maintained only in VA2 (Figure 5B and 5D), we identified two VA2 muscles (that co-express *Poxm* and *Kr*) and two *Poxm*-expressing VA1 muscles in late stage 14 *Df(3L)iro^DFM3^* embryos (Figure 5F). The presence of the duplicated VA1 and VA2 muscles was more evident at stage 15 when *Poxm* was only weakly expressed in VA2 muscles (Figure 5G). We concluded that Ara and Caup were required to specify LT progenitors and that implementation of this fate implies the repression of specific muscle identity genes, such as *slou* in P_LT3/4_ and *vg* in P_LT1/2_. Moreover, it seemed that the only muscles affected by the lack of *ara/caup* were those in which these genes were already expressed in the corresponding promuscular clusters, since the fate of DT1 and SBM, visualised by the expression of *slou*, *Con* and *lb*, was apparently unaffected in *Df(3L)iro^DFM3^* embryos (Figure 2F, Figure 5D and 5E, and Figure S1E–S1H).

### Ras/MAPK cascade modulates the regulation of *slou* by Caup in Schneider-2 cells

**Figure 6:**
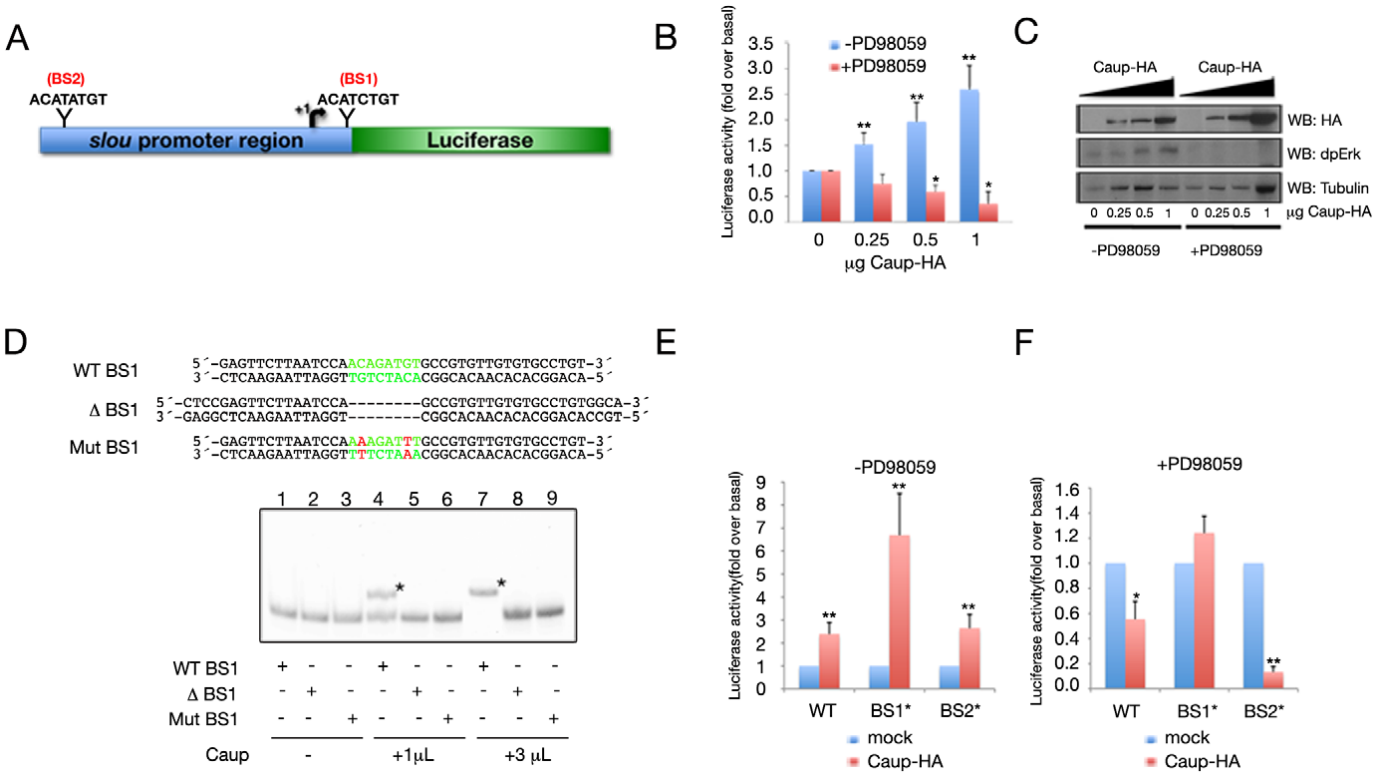
Direct interaction of Caup with *slou* regulatory region and its modulation by the Ras/MAPK pathway. (A) Diagram of the 2 Kb long *slou* promoter region (from −1828 to +153 nt) used to drive Luciferase expression. This fragment contains two putative binding sites for Ara/Caup, BS1 and BS2. (B) Effect of increasing amounts of Caup-HA on the Luciferase activity driven by *slou* promoter in the absence (blue bars) and presence (red bars) of PD98059 MEK1 inhibitor. (C) Representative western blots of lysates of S2 cells expressing increasing amounts of Caup (upper panel) in the absence and presence of PD98059, showing the state of activation of the Ras/MAPK cascade (middle panel) and Tubulin expression as loading control (lower panel). (D–F) Mutagenesis analysis of *slou* regulatory region. (D) Binding of Caup to the indicated *slou* regulatory fragments, containing BS1 determined by EMSA. Binding of Caup to wild-type fragment resulted in the formation of complexes with reduced mobility (asterisk in lane 4), which was more evident in the presence of increased amounts of Caup (asterisk in lane 7). No shift was observed when fragments devoid of BS1 (Δ BS1, lanes 5, 8) or point-mutated (Mut BS1, lanes 6, 9) were used or in the absence of Caup (lanes 1–3). (E, F) Effect of Caup-HA (1 µg) on the Luciferase activity driven by wt and mutated (BS1*, BS2*) *slou* promoter regions in the absence (E) and presence (F) of PD98059 inhibitor. Statistical analyses for Luciferase assays were performed using the paired two-tailed Student's t-test. The data are presented as means ± S.E.M. of 3 independent experiments. *P<0.05, **P<0.001 compared to basal (B) or wt (E, F) conditions.

Our data suggested that Ara/Caup might act as repressors of *slou* in the *Drosophila* mesoderm. Therefore we decided to investigate whether *slou* might be a direct target of Ara/Caup. An “in silico” search of a previously reported *slou* cis-regulatory region [46] identified two putative Iro binding sites (BS) at positions +129 (BS1) and −1642 (BS2), relative to the transcription start site, which match the consensus ACAN_2–8_TGT ([47] and Figure 6A). We cloned this regulatory region in a Luciferase reporter vector and measured Luciferase activity in *Drosophila* Schneider-2 (S2) cells transiently transfected with this construct and increasing amounts of HA-tagged Caup. Contrary to expectations, we found that addition of Caup-HA increased the basal Luciferase activity driven by the *slou* regulatory region in a dose dependent manner (blue bars in Figure 6B), indicating that Caup acts as a transcriptional activator of *slou* under these conditions. The reported regulation of the chicken Irx2 factor by MAPK (that switches it from repressor to activator) could explain this result [48]. Since Western Blot analysis of S2 lysates using an antibody against diphospho-extracellular-signal related kinase (dpErk) showed the MAPK pathway to be active in S2 cells (Figure 6C) and we have obtained experimental evidence showing the presence of phosphorylated Caup in S2 cells with constitutively active MAPK pathway (N.B, A.S.T and S.C, manuscript in preparation), we hypothesized that the activation effect of Caup in S2 cells could be due to the Ras/MAPK cascade turning Caup from transcriptional repressor into activator. Indeed, the inhibition of the Ras/MAPK pathway by the PD98059 MAP-erk kinase-1 (MEK1) inhibitor induced a Caup-dose dependent decrease in Luciferase activity driven by the *slou* regulatory sequences (Figure 6B, red bars). This result could not be attributed to a direct effect of the inhibitor over the *slou* promoter, since its addition did not modify the basal Luciferase activity of the construct (Figure 6B).

To test whether Caup-dependent transcriptional regulation relied on a direct interaction of Caup with the *slou* regulatory region we performed electrophoretic mobility shift assays (EMSA) with in vitro translated Caup and wild-type and mutated Caup-BS. These assays indicate efficient binding of Caup to BS1, which is abolished by BS1 mutation and deletion (Figure 6D). In contrast, Caup appears not to bind BS2 under these experimental conditions (not shown).

Next we examined the functional relevance of BS1 and BS2 in the Luciferase reporter assay. Deletion of BS2 had no major effect on Caup-dependent *luciferase* expression compared to the wild-type promoter (Figure 6E and 6F compare with Figure 6B). This result suggested that Caup does not bind to BS2 (as indicated by the EMSA data). Unexpectedly, deletion of BS1 resulted in a more efficient activation of *luciferase* expression than that driven by the wild type regulatory region (Figure 6E). This suggested that binding of Caup to BS1 somehow impaired transcription. Note that the activation of *luciferase* driven by the BS1 mutated regulatory region was still dependent on the MAPK pathway (Figure 6E and 6F). This suggests that such activation appears to depend on the binding of a MAPK-dependent phosphorylated protein, which we hypothesize might be Caup, to a so far unidentified binding site. Thus, the analysis in S2 cells confirmed the relevance of BS1, but not of BS2 on Caup-dependent regulation.

Additionally, we have analysed the evolutionary conservation of these putative Caup-BS among several *Drosophila* species (Figure S3). Notably, BS1 is located in a highly conserved region and its sequence is identical across the melanogaster group, whereas neither BS2 nor the adjacent sequences are conserved. These data further reinforce the relevance of BS1 for Caup-dependent *slou* regulation.

Our results are thus consistent with a direct effect of Caup on *slou* regulation. However, it cannot be ruled out the possibility of the existence in vivo of a transcription factor, acting downstream of *ara/caup*, that could repress *slou* through BS1 or through a still unidentified regulatory sequence of *slou*.

### Caup integrates in vivo inputs from the Ras/MAPK cascade for its regulation of *slou*

**Figure 7:**
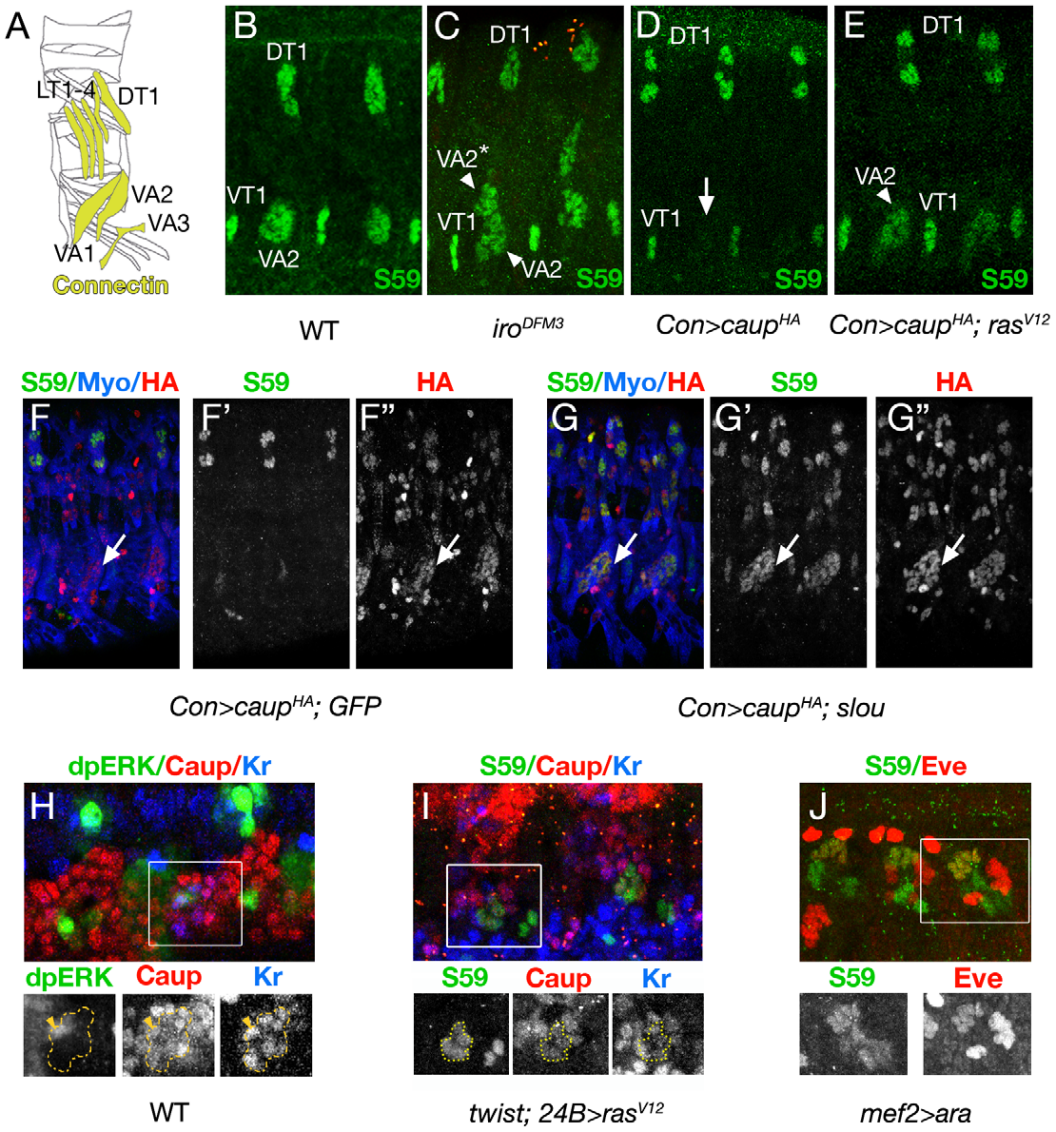
Ras/MAPK modulates the transcriptional activity of Caup on *slou* during myogenesis. (A) Schematic drawing of muscles expressing *Con* in abdominal hemisegments. (B–E) Lateral views of abdominal hemisegments of stage 15–16 wild type (B), *Df(3L)iro^DFM3^* (C), *Con-GAL4::UAS-caup^HA^* (D) and *Con-GAL4::UAS-caupHA; UAS-ras^V12^* (E) embryos, stained with S59 antibody. Note the presence of an ectopic VA2 muscle (VA2*) in *Df(3L)iro^DFM3^* (C), the absence of *slou* in VA2 when *caup* is ectopically expressed in this muscle (arrow, D, see also F-F″), and the failure of Caup to repress *slou/*S59 in VA2 muscle in the presence of the activated form of Ras, *ras^V12^* (E). (F-G″) Lateral views of stage 15–16 *Con-GAL4::UAS-caupHA; UAS-GFP* (F-F″) and *Con-GAL4::UAS-caupHA; UAS-slou* (G-G″) embryos stained with the indicated antibodies. Note that co-expression of *caup* and *slou* in VA2 does not appreciably modify the morphology of the muscle (arrows in G-G″). As an internal control co-expression of *UAS-caup* and *UAS-GFP* still repressed endogenous *slou* and prevented the VA2 fate (F-F″). (H) Close-up of a lateral transverse promuscular cluster (outlined) in a stage 11 wild-type embryo showing co-expression of Caup (red) and Kr (blue) in all cells of the clusters. Note that the activation of the Ras/MAPK cascade (dpErk, green) only takes place at low levels in the segregating progenitor (yellow arrowhead) but not in the rest of the cluster. (I) Close-up of LT cluster in *twist-GAL4; 24B-GAL4::UAS- ras^V12^* stage 11 embryo. Early activation of the Ras/MAPK pathway prevents the repression of *slou* by Caup in the LT cluster. (J) Close-up of the dorsal mesoderm of a *mef2-GAL4::UAS-ara* stage 15 embryo showing ectopic expression of *slou* in *eve*-expressing muscles.

To further examine in vivo the regulatory activity of Caup on *slou* (Figure 7B, 7C), we ectopically expressed *caup* or *ara* in VA1–3 using *Con-GAL4* and checked whether they would repress *slou* in the VA2 muscle. This was indeed the case (Figure 7B, 7D, 7F-7F″ and not shown). Loss of *slou* expression caused by ectopic *caup* reproduced the morphological defects in VA2 previously described in *slou* mutants (Figure 7F-7F″ and [17]). To analyse whether the morphological effect of Caup on muscle VA2 development was only due to Caup-dependent repression of *slou*, we forced the expression of both genes using the *Con-GAL4* driver. In this experimental condition Caup was unable to repress *UAS-slou* expression and the VA2 muscle and its morphology seemed unaffected (Figure 7F-7G″).

Once verified the repressor activity of Caup on *slou* during myogenesis, to analyse the regulatory potential of BS1 in vivo we generated transgenic flies harbouring the wild-type or the BS1 deleted version of the *slou* regulatory region. The wild-type regulatory region only partially reproduced the *slou* endogenous expression, as it drove *lacZ* expression in the CNS but not in the muscles (not shown and Figure S4). In contrast, the construct lacking BS1 behaved congruently with our S2 cells results, since it drove ectopic expression of *lacZ* in the lateral muscles (Figure S4). Curiously, up-regulation of *lacZ* was found in the 4 lateral muscles and not only in the ones that show *slou* expression in the absence of Ara/Caup (Figure 4B). Thus we interpret that this construct, while missing some of the regulatory sequences required for *slou* mesodermal expression, it contains those required for Caup mediated repression in the mesoderm. In addition, the absence of strict correlation between the phenotypes of deletion of BS1 and lack of Ara/Caup, might indicate the ability of other transcription factor(s) to regulate *slou* expression in LT1–2 through BS1.

To investigate whether the effect of the MAPK cascade on the transcriptional activity of Ara/Caup found in the S2 cell assay is also at work during myogenesis we examined whether there is a correlation between MAPK signalling and Caup transcriptional regulatory activity. We looked at the state of activation of this pathway in the LT promuscular cluster, where Ara/Caup repress *slou*, and found that it did not appreciably express dpErk (Figure 7H). Therefore, a repressor activity of Ara/Caup correlates in vivo with the absence of MAPK signalling. Next, we tested whether forced activation of the MAPK pathway in the mesoderm could interfere with the repressor activity of endogenous Caup in LT promuscular clusters. This was indeed the case, since activation of the MAPK pathway using *twist-GAL4*; *24B-GAL4* to drive the activated form of Ras85D (*ras^V12^* [49]) allowed co-expression of *caup* and *slou* in this cluster (Figure 7I). Similarly, late co-expression of *ras^V12^* and *caup* (*Con-Gal4* driver) blocked the repression activity of Caup on *slou* (Figure 7D and 7E). Finally, to test whether MAPK signalling not only prevented Caup-dependent repression of *slou* but also converted Caup from repressor to activator, we looked at the expression of *slou* after early pan-mesodermal *Caup* expression (*mef2-GAL4*). As shown in Figure 7J, Ara was indeed able to ectopically activate *slou* in *Drosophila* epidermal growth factor receptor (DER)-dependent *eve*-expressing muscles.

## Discussion

The study of myogenesis in *Drosophila* has increased the understanding of how the mechanisms that underlie the acquisition of specific properties by individual muscles are integrated within the myogenic terminal differentiation pathway. Thus, the current hypothesis proposes that distinct combinations of regulatory inputs leads to the activation of specific sets of muscle identity genes in progenitors that regulate the expression of a battery of downstream target genes responsible for executing the different developmental programmes (reviewed in [2], [10], [38]). However, the analysis of the specific role of individual muscle identity genes and of their hierarchical relationships is far from complete since the characterisation of direct targets for these transcriptional regulators is very scarce [36], [37].

Here, we report the identification of *ara* and *caup*, two members of the Iroquois complex, as novel type III muscle identity genes. We find that the homeodomain-containing Ara and Caup proteins are necessary for the specification of the LT fate. *ara/caup* appear to be bona fide muscle identity genes. Indeed, similarly to the identity genes *Kr* and *slou* [17], [18], absence of *ara/caup* does not interfere with the segregation of muscle progenitors or their terminal differentiation, but modifies the specific characteristics of LT1–4 muscles, which are transformed towards VA1, VA2, LL1 and LL1 sib fates. These transformations may be due in part to the up-regulation of *slou* and *vg* in the corresponding muscles. Thus, a recent report [50] shows that forced expression of *vg* in LT muscles induces changes in muscle attachments similar to the ones observed in LT1 in *ara/caup* mutant embryos. However, it should be stressed that although in *ara/caup* mutants LT muscles are lost in more than 95% of cases, they are not completely transformed into perfect duplicates of the newly acquired fates. For instance, while the specific LT marker *lms* is lost in 91% of cases, ectopic *slou* expression is detected in only 75% of cases. These partial transformations might be due to differences in the signalling inputs acting in the mesodermal region from where these muscles segregate (see below). Our unpublished data also showed that forced pan-mesodermal expression of *ara/caup* alter the fates of many muscles both in dorsal and in ventral regions without converting them into LT muscles (i.e., they do not ectopically express *lms*). Similarly, *Kr* and *slou* ectopic expression is not sufficient to implement a certain muscle fate [17], [18]. The failure to recreate a given muscle identity by adding just one of the relevant muscle identity proteins reveals the importance that cell context, that is, the specific combination of signalling inputs and gene regulators present in each cell, have in determining a specific muscle identity.

Our analysis of the myogenic requirement of *ara/caup* has revealed several features about how these genes act to implement LT fates. Thus, although they are expressed in six developing embryonic muscles, only four of them, LT1–4, are miss-specified in the absence of Ara/Caup. The remaining two, DT1 and SBM, seem to develop correctly, according to morphological as well as molecular criteria. It is worth noting that the requirement for *ara/caup* genes in these six muscles correlates with the onset of their expression. Thus, in the affected LT1–4 muscles Ara/Caup can be first detected at the earliest step of muscle lineages, that is in the promuscular clusters. In contrast, in the unaffected muscles *ara/caup* start to be expressed later, in the DT1/DO3 progenitor and the SBM founder. This suggests that in muscle lineages *ara/caup* have to be expressed very early to repress *slou* and *vg* to implement the LT fate. Several data support this interpretation. For instance, the observation that *ara/caup* are co-expressed with *slou* in DT1, whereas they repress *slou* in LT3–4, may be related to the fact that *slou* expression precedes that of *ara/caup* in the DT1 lineage. Should this be so, one would expect that ectopic expression of *ara* using the early driver *mef2-GAL4*, would repress *slou* in DT1, as it actually does (Figure S5), whereas this repression is not evident using the late driver *Con-GAL4*. Furthermore, the hypothesis of the relevance of the timing of muscle identity gene expression for muscle fate specification might also apply to the case of *slou*, where a similar correlation between the strength of the loss-of-function *slou* phenotypes in specific muscles and the onset of *slou* expression has also been found [17].

**Figure 8:**
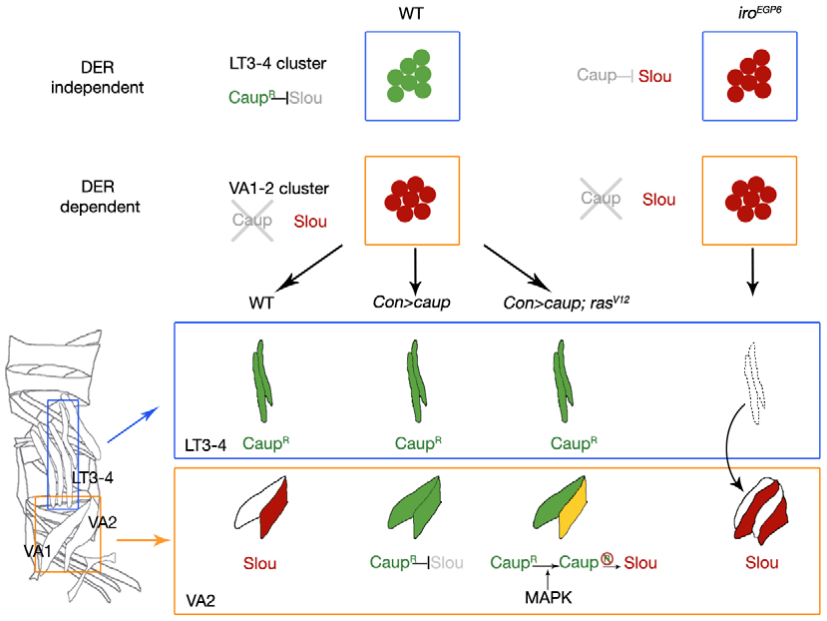
Effect of the state of activation of the Ras/MAPK signalling cascade on the regulation of *slou* by Ara/Caup in LT and VA lineages. In the wild-type LT3–4 promuscular cluster, where Ras/MAPK signalling is inactive, Caup represses *slou* since in embryos mutant for *ara/caup* (*Df(3L)iro^EGP6^*), the absence of Caup allows *slou* activation in this cluster and the consequent transformation of LT3–4 muscles to VA1–2 muscles. In the wild-type Caup is absent from the DER-dependent VA1–2 cluster that expresses *slou*. Ectopic expression of Caup in the VA1–2 lineages using *Con-GAL4* (active after founder segregation when MAPK signalling is extinguished) represses *slou* in VA2. On the contrary, *Con-GAL4* driven expression of Caup together with the activated form of Ras alleviates Caup-dependent *slou* repression in the VA2 muscle.

It should be stressed that the generation of the LT code depends not only on the early presence of Ara/Caup on the promuscular clusters but also on the absence (or strong reduction) of DER/Ras activity at that precise developmental stage and location (Figure 8). There is a dynamic regulation of MAPK signalling in the lateral mesoderm. Caup-expressing muscles develop from DER-independent clusters whereas the duplicated muscles observed in *ara/caup* mutants derive from progenitors that segregate very near the LT progenitors [3], but originate in DER-dependent promuscular clusters that are specified slightly later in development [4], [51]. Furthermore we have observed both by in vivo and in cell culture that low MAPK activity is required for Caup-dependent *slou* repression. Therefore, we interpret the role of Ara/Caup in the implementation of LT fate as follows (Figure 8). At mid stage 11 in the myogenic mesoderm, groups of mesodermal cells acquire myogenic competence as a result of interpreting a combinatorial signalling code that reflects their position along the main body axes, as well as the state of activation of different signalling pathways [4]. Accordingly, these clusters initiate the expression of *lethal of scute* and a unique code of muscle identity genes, as has been shown in great detail for *eve* expression in the dorsal mesoderm [34], [35]. In the case of the dorso-lateral mesoderm this code includes *ara/caup* and *Kr* and implements the LT fate. Since the level of activation of the Ras/MAPK cascade is low in these clusters, Ara/Caup will behave as transcriptional repressors, preventing the activation of *slou* or *vg* in LT1–2 and LT3–4 clusters, which would be otherwise activated in this location. Thus, Ara/Caup implement the LT fate by repressing the execution of the alternative fates (Kr+, Slou+, Con+, Poxm+ and Kr+, Vg+) that would give rise to duplicates of P_VA1/VA2_ and P_LL1/LL1sib_, respectively, and by allowing a different identity gene code (Kr+, Caup+, Con+, *lms*+) that generates the LT fate.

Slightly later the Ras/MAPK pathway becomes active at the dorsolateral region (Figure 8). This changes the combinatorial signalling code and coincides with a change in the muscle identity genes expressed by the promuscular clusters that segregate from this position, which now accumulate Kr but not Ara/Caup. Progenitors born from them will express either *slou* or *vg* and give rise to VA1–2 and LL1/LL1sib fates, all DER-dependent [51].

Our S2 cells experiments suggest a molecular mechanism by which the Ras/MAPK pathway modulates the transcriptional activity of Ara/Caup on *slou*. Thus, low MAPK activity and direct binding of Caup to BS1 site of the *slou* gene would favour strong repression of *slou*. BS1 could be embedded in a silencer regulatory element or its binding to Caup may block transcription of the downstream located *luciferase* gene. On the contrary, Caup-dependent activation of *slou* would be dependent on MAPK signalling. We hypothesize that MAPK–dependent Caup phosphorylation could modulate its interaction with different transcriptional co-factors or/and its binding site affinity.

Furthermore, our in vivo evidence indicates a repressor function of presumably non-phosphorylated Caup on *slou* since forced activation of the Ras pathway allows co-expression of *slou* and *caup*. On the other hand, the ectopic expression of *slou* induced by *caup*-over-expression is suggestive of a possible activator function of phosphorylated Caup.

The role of IRO proteins in cell fate specification is conserved in both vertebrates and invertebrates (reviewed in [52]). Here we have shown that the interplay between MAPK signalling and IRO activity found in vertebrate neuroepithelium [48] is also at work in *Drosophila* myogenesis. We have also identified a potential direct target of Ara/Caup, *slou* and propose *vg* as a candidate gene to be regulated by Ara/Caup. In both cases the genes subordinated to *ara/caup* encode transcription factors that might in turn regulate the expression of other genes, genes that must be repressed in LT muscles in order to acquire the LT fate. These results, therefore, provide insights into the way Ara/Caup control lateral muscle identity and on the role of signalling pathway inputs to modulate the activity of these transcription factors, with consequences in their downstream targets. It also highlights the importance that the specific combination of muscle identity genes, their hierarchical relationships and their temporal activation have in determining the identity of a given muscle cell, very alike to what is at work during the acquisition of neural fates [53].

## Materials and Methods

### *Drosophila* stocks

The following stocks were used: *Df(3L)iro^DFM3^*, *ara^rF209^* [28], *Df(2L)5* [54], *Df(3L)iro^EGP6^*, *Df(3L)iro^EGP5^*, *Df(3L)iro^EGPΔ1^* [42], *rP298* [32], *mef2-GAL4* [55], *Con-GAL4* [56], *twist-GAL4; 24B-GAL-4* (a gift from M. Baylies), *UAS-ara, UAS-caup* [28], *UAS-caup-HA* (N. Barrios, unpublished) and *UAS-ras^V12^* [49]. Ectopic expression was generated by means of the GAL4/UAS system [57].

### In situ hybridisation, immunohistochemistry, and microscopy

Whole-mount in situ hybridisation with digoxygenin-labelled RNA probes and immunocytochemistry were performed as described previously [58]. Stained embryos were embedded in Araldite and sectioned (3 µm) following standard procedures. The following primary antibodies were used at the indicated dilutions: rat anti-Caup (1∶50) [23], guinea pig anti-Kr (1∶500) [59], mouse anti-Lb (1∶1) [15], rabbit anti-Poxm (1∶10) [14], rat and rabbit S59 (that recognises Slou, 1∶50) [3], rabbit anti-Alien (1∶500) [60], mouse anti-Con (1∶10) [61], rabbit anti-Vg (1∶500) [62], rat-anti- Tropomyosin (MAC141; 1∶100; Babraham Tech), rabbit anti-Myosin (Myo; 1∶300) [63], rat anti-HA (1∶1000; Roche); rabbit anti-ß-Gal (1∶5000; Cappel) and mouse anti- dpErk (1∶50; Sigma). Images were obtained with confocal microscopes MicroRadiance (BioRad) and LSM510META (Zeiss) and analysed using the software Zeiss LSM Image or LaserSharp and Adobe Photoshop 7.0. In most cases the figures correspond to z-projections of series of confocal sections.

### Cell culture and transfections

The 5′-upstream region of *slou* (from −1828 to +153 nt) was amplified via PCR and cloned in pGLHS43 vector, a modified version of the pGL2-basic vector (Luciferase reporter plasmid, Promega), obtained after substitution of the SV40 promoter by the *Drosophila heat-shock 43* minimal promoter (a gift from A. Baonza). The putative Caup BS1 and BS2 were deleted using the “Quick Change” site-directed mutagenesis kit (Stratagene, SantaClara, CA). The sequences of the primers used to delete BS1 were 5′-GAGTTCTTAATCCAGCCGTGTTGTGTGCCTGTGGCAAGTCAATAG-3′ and its reverse complement and for BS2, 5′-CCATATACATATGTGTGCATGTATGCATAAGTGTGAGTGTGAGTGGG -3′ and its reverse complement. pAC5.1-Caup-HA plasmid was obtained after cloning *caup* ORF with an HA tag in the *Drosophila* expression vector pAC5.1 (Invitrogen). *Drosophila* S2 cells were cultured in Insect-Xpress medium (Lonza) supplemented with 7% fetal bovine serum and grown at 25°C. For Luciferase assays S2 cells were seeded at a density of 2×10^6^ and co-transfected with 1 µg of the different firefly Luciferase reporter constructs DNA, 30 ng of control plasmid (expressing Renilla Luciferase driven by the promoter of Drosophila RpIII128, [64]) and either 0, 0.25, 0.5 or 1 µg of pAC5.1-Caupolican-HA plasmid per well using Nucleofector Technology (Lonza). Luciferase activity in the cell extracts was measured using Dual-Glo Luciferase assay system (Promega) following the manufacturer's protocol. Briefly, 20 µl extract was added to 100 µl F-luc assay reagent, mixed gently for 5 s and placed in a luminometer. After counting F-luc activity for 10 s, 100 µl stop-and-glo reagent was added to the tube, mixed gently for 5 s and placed in the luminometer for R-luc count. The R-luc activities were used as internal control to correct for the difference in transfection efficiency of different reporter plasmids. Therefore, F-Luc/R-Luc activities were used for data analysis. To investigate whether the MEK/ERK pathway was involved in transcriptional regulation driven by the *slou* promoter, S2 cells were treated or not with 50 µM PD-98059 (Sigma) for 2 hrs before Luciferase activity measurement. All data reported are means from three or four independent experiments, each performed in triplicates. Primary antibodies used in immunoblots were mouse anti-dpErk (1 µg/ml; Sigma), rat anti-HA (200 ng/ml; Roche) and mouse anti-βtubuline (1∶5000; Developmental Studies Hybridoma Bank).

### Generation of *slou* reporter transgenic lines

The 5′-upstream region of *slou* used in S2 cells in the Luciferase reporter assays (both the wild type sequence and that missing the putative Caup BS1) were subcloned at the EcoRI site of the C4PLZ enhancer tester plasmid that contains a weak P-element promoter [65]. These *lacZ* reporter plasmids were introduced into *y w^1118^* embryos by standard P-element transformation.

### Electrophoretic mobility shift assay

Caup binding ability to the *slou* promoter region was analyzed by EMSA. Pairs of single-stranded, Cyc3 and unlabeled 40-mer oligonucleotides containing the wild-type putative Caup binding sites BS1, BS2 and their mutant or deleted versions were allowed to anneal to generate double-stranded probes. Sequences of primers are shown in Figure 6D for BS1 and in Dataset S1. Caup protein was synthesized *in vitro* by using the coupled transcription/translation rabbit reticulocyte lysate system (TNT Promega). The indicated amount of µl of TNT reaction mixture was incubated with 20 ng of labelled probe. Protein–DNA complexes were allowed to form at room temperature for 30 min in a total volume of 20 µl of binding buffer (50 mM HEPES, pH 7.5, 10 mM MgCl_2_, 10 mM KCl and 1 mM DTT). After incubation, free DNA and protein–DNA complexes were resolved by 6% non-denaturing polyacrylamide gel electrophoresis. Gel fluorescence was analyzed in a Typhoon Scanner (GE healthcare).

## Supporting Information

Dataset S1 Sequences of primers used in EMSA to analyse binding of Caup to BS2. Pairs of 40-mer oligonucleotides containing the wild-type putative Caup binding sites BS2 and their mutant or deleted versions are shown.
(DOCX)

Figure S1 Pattern of expression of muscle marker genes in *Df(3L)iro^DFM3^* embryos. (A–D) RNA in situ hybridisation with *lms* probes of stage 13 (A, B) and stage 15 (C, D) *yw* (A, C) and *Df(3L)iro^DFM3^* (B, D) embryos, showing the normal early onset of *lms* expression in the lateral region of abdominal segments in the mutant embryos (B, compare to A) and its absence of expression at later stages (D, compare to C). (E, F) Lateral view of stage 14 *yw* (E) and *Df(3L)iro^DFM3^* (F) embryos stained with anti-Con antibodies, showing the absence of *Con*-expressing lateral muscles (asterisk in F) and the presence of *Con*-expressing DT1, VA2 and ectopic VA2 (VA2* in F) in *Df(3L)iro^DFM3^* embryos (F, compare to E). (G, H) Lateral view of stage 15 *yw* (G) and *Df(3L)iro^DFM3^* (H) embryos stained with anti-Lb antibodies to show the presence of *lb*-expressing SMB in *Df(3L)iro^DFM3^* embryos (H, compare to G).
(TIF)

Figure S2 Regulation of *caup* expression during embryogenesis. (A, B) Lateral view of stage 15 wild-type (A) and *Df(2L)5* (B) embryos stained with anti-Alien (green) and anti-Caup (red). Note that in *Df(2L)5* embryos despite the absence of Caup ectodermal expression (asterisk in A), apodema specification (labelled by Alien) and Caup mesodermal expression (arrowheads) are indistinguishable from wild-type embryos.
(TIF)

Figure S3 Caup BS1 but not BS2 of *slou* cis-regulatory region is evolutionary conserved between *Drosophila* species in the *melanogaster* group. The *slou* cis-regulatory region used in this study was compared between drosophilids using the VISTA Browser tool of VISTA tools for comparative genomics (http://genome.lbl.gov/vista/index.shtml). We found a high degree of similarity in this region between *D. melanogaster* and other members of the melanogaster subgroup (*D. simulans*, *D. yakuba* and *D. erecta*) and only partial similarity with more distant species like *D. ananassae* (melanogaster group) and *D. pseudoobscura* (obscura group). BS1 is located in a highly conserved region and its sequence is identical across the melanogaster group, whereas BS2 is located in a region of low conservation and not found in any of the related species. Significant similarities on *slou* coding and cis regulatory regions were only found between *Drosophila melanogaster* and the closer drosophilid species *D. simulans*, *D. yakuba*, *D. erecta* and *D. ananassae*. No homology was found using the BLAST tool (http://blast.ncbi.nlm.nih.gov/Blast.cgi) with *Anopheles gambie*, *Apis mellifera*, *Xenopus tropicalis*, *Danio rerio*, *Mus musculus* and *Homo sapiens*.
(TIF)

Figure S4 Deletion of Caup BS1 promotes *lac-Z* expression in LT muscles driven by *slou* cis-regulatory region. Lateral views of stage 15 *slou-lacZ* (A, A′) and *ΔBS1-slou-lacZ* (B, B′) embryos stained with anti-Caup (green), anti-ßgal (red) and anti-Myo (blue) antibodies. Note absence of *lacZ* expression in LT muscles of *slou-lacZ* embryos (arrows in A, A′) and co-expression of *caup* and *lacZ* in LT muscles of *ΔBS1-slou-lacZ* embryos (arrows in B, B′).
(TIF)

Figure S5 Repression of *slou* by ectopic expression of Ara. Lateral views of stage 15 wild-type (A) and *mef2-GAL4::UAS-ara* (B-B′) embryos stained with anti- Tropomyosin (red) and anti-slou (green) antibodies. (A) Note *slou* expression in DT1, VA2 and VT1 muscles (arrows). (B) Early expression of *ara* with the panmesodermal driver *mef2-GAL4* represses *slou* in DT1, VA2 and VT1 in many segments (arrows). A few muscles maintain *slou* expression (asterisks).
(TIF)
