# Supplementary material for: Drosophila Araucan and Caupolican Integrate Intrinsic and Signalling Inputs for the Acquisition by Muscle Progenitors of the Lateral Transverse Fate
Source: PLoS Genet. 2011 Jul 21;7(7):e1002186. doi: 10.1371/journal.pgen.1002186 (PMC3141015; doi:10.1371/journal.pgen.1002186)
Supplement: Dataset S1 — Sequences of primers used in EMSA to analyse binding of Caup to BS2. Pairs of 40-mer oligonucleotides containing the wild-type putative Caup binding sites BS2 and their mutant or deleted versions are shown. (DOCX) [file pgen.1002186.s001.docx]

GAATGTCCATATACATACATATGTATGTGTGCATGTATGC WT BS2 5’-3’

GCATACATGCACACATACATATGTATGTATATGGACATTC WT BS2 5’-3’

GAATGTCCATATACATAAATATTTATGTGTGCATGTATGC Mut BS2 5’-3’

GCATACATGCACACATAAATATTTATGTATATGGACATTC Mut BS2 5’-3’

ATAGAATGTCCATATACATATGTGTGCATGTATGCATAAG ∆ BS2 5’-3’

CTTATGCATACATGCACACATATGTATATGGACATTCTAT ∆ BS2 5’-3’
