# Supplementary figures and images for: Drosophila Araucan and Caupolican Integrate Intrinsic and Signalling Inputs for the Acquisition by Muscle Progenitors of the Lateral Transverse Fate
Source: PLoS Genet. 2011 Jul 21;7(7):e1002186. doi: 10.1371/journal.pgen.1002186 (PMC3141015; doi:10.1371/journal.pgen.1002186)

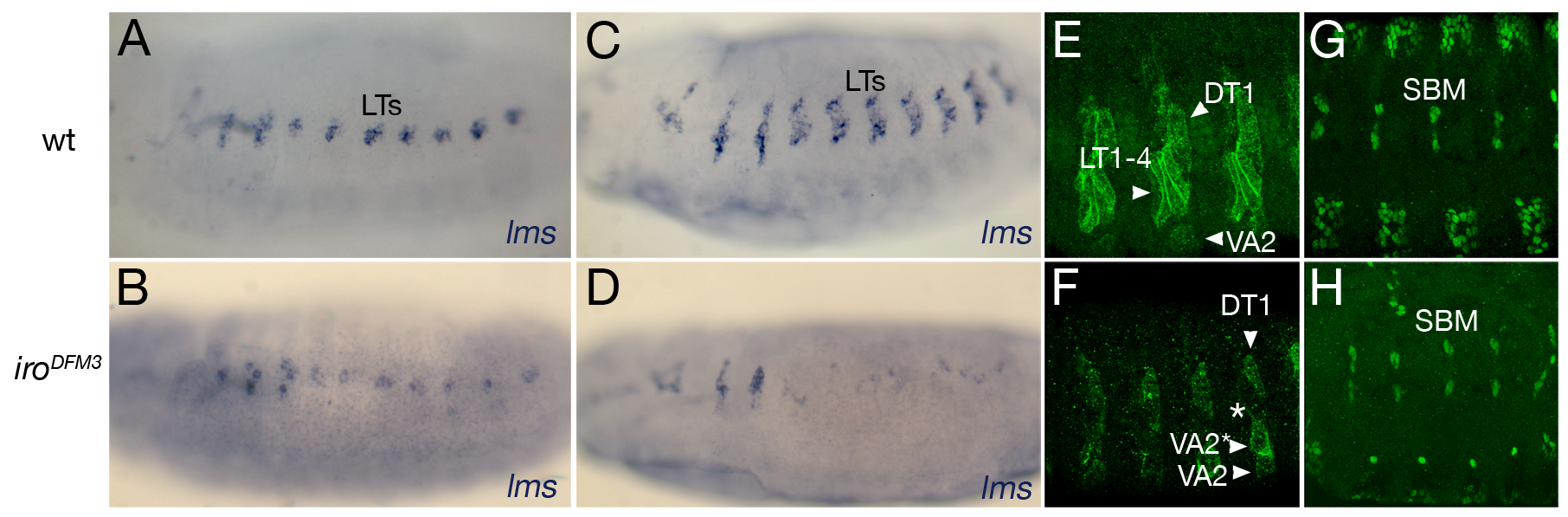

Supplement: Figure S1 — Pattern of expression of muscle marker genes in Df(3L)iroDFM3 embryos. (A–D) RNA in situ hybridisation with lms probes of stage 13 (A, B) and stage 15 (C, D) yw (A, C) and Df(3L)iroDFM3 (B, D) embryos, showing the normal early onset of lms expression in the lateral region of abdominal segments in the mutant embryos (B, compare to A) and its absence of expression at later stages (D, compare to C). (E, F) Lateral view of stage 14 yw (E) and Df(3L)iroDFM3 (F) embryos stained with anti-Con antibodies, showing the absence of Con-expressing lateral muscles (asterisk in F) and the presence of Con-expressing DT1, VA2 and ectopic VA2 (VA2* in F) in Df(3L)iroDFM3 embryos (F, compare to E). (G, H) Lateral view of stage 15 yw (G) and Df(3L)iroDFM3 (H) embryos stained with anti-Lb antibodies to show the presence of lb-expressing SMB in Df(3L)iroDFM3 embryos (H, compare to G). (TIF) [file pgen.1002186.s002.tif]

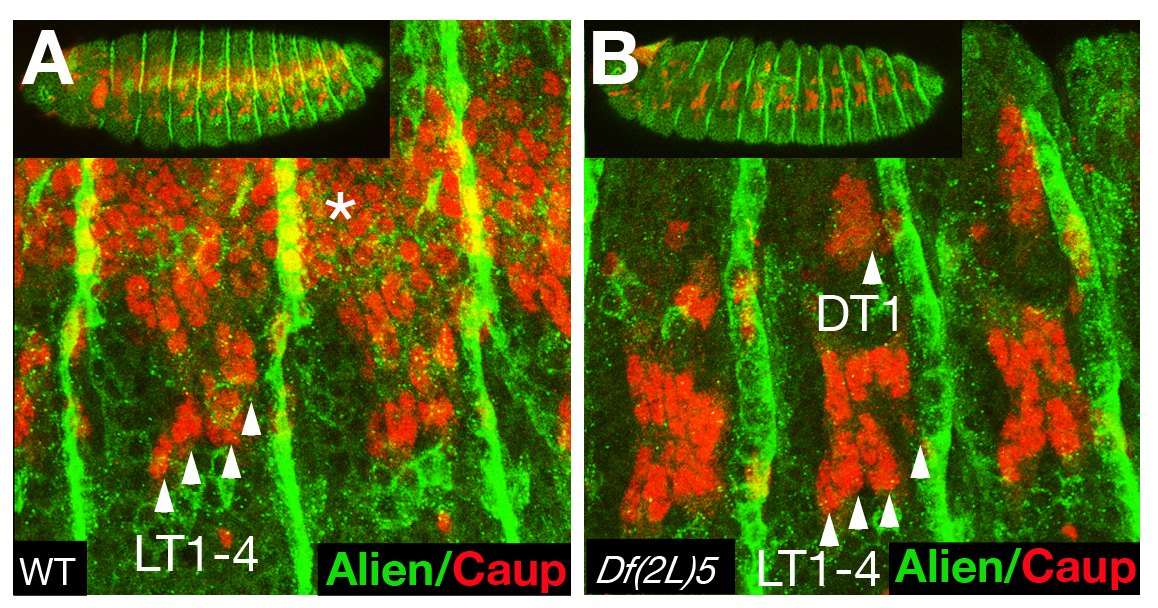

Supplement: Figure S2 — Regulation of caup expression during embryogenesis. (A, B) Lateral view of stage 15 wild-type (A) and Df(2L)5 (B) embryos stained with anti-Alien (green) and anti-Caup (red). Note that in Df(2L)5 embryos despite the absence of Caup ectodermal expression (asterisk in A), apodema specification (labelled by Alien) and Caup mesodermal expression (arrowheads) are indistinguishable from wild-type embryos. (TIF) [file pgen.1002186.s003.tif]

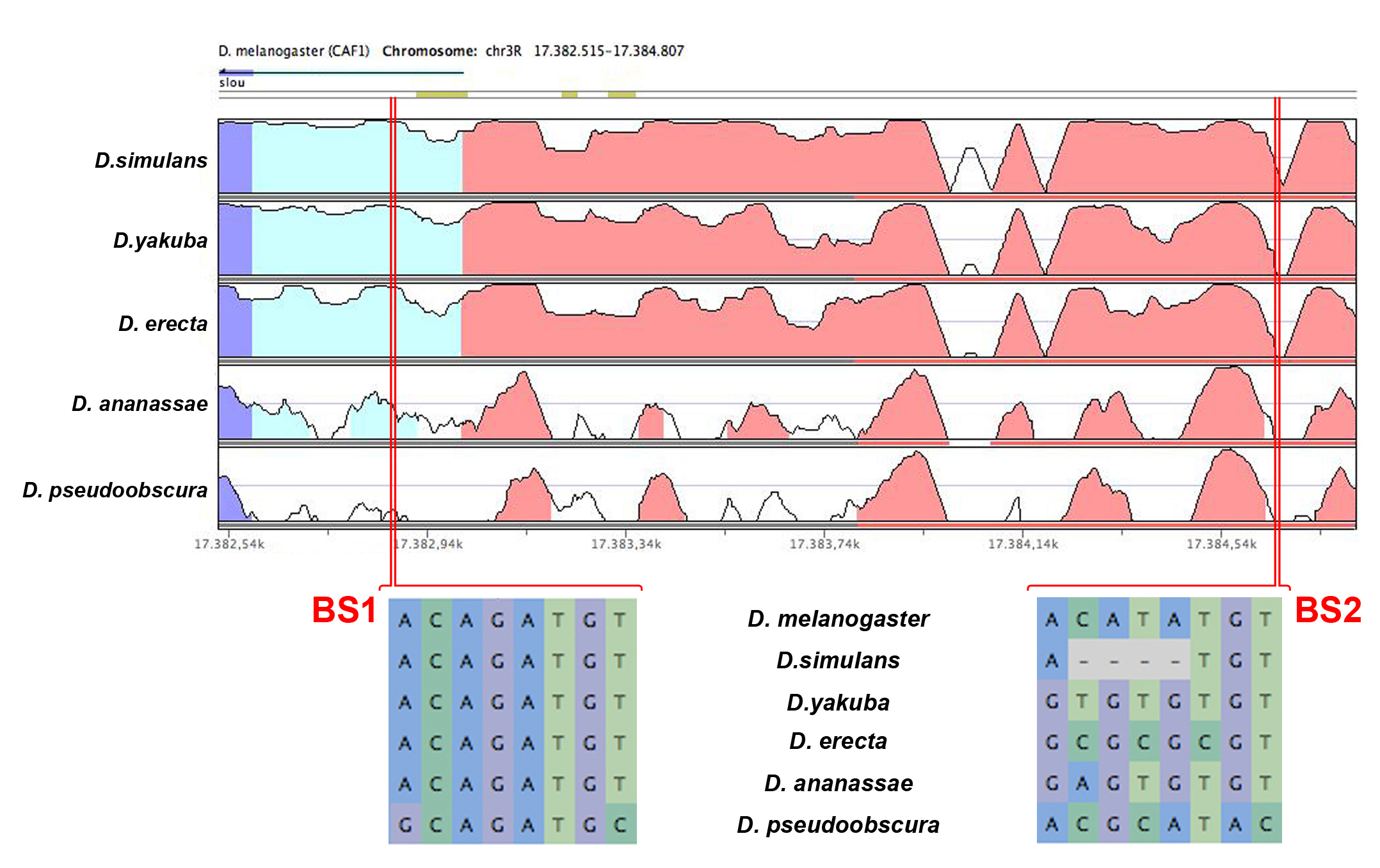

Supplement: Figure S3 — Caup BS1 but not BS2 of slou cis-regulatory region is evolutionary conserved between Drosophila species in the melanogaster group. The slou cis-regulatory region used in this study was compared between drosophilids using the VISTA Browser tool of VISTA tools for comparative genomics (http://genome.lbl.gov/vista/index.shtml). We found a high degree of similarity in this region between D. melanogaster and other members of the melanogaster subgroup (D. simulans, D. yakuba and D. erecta) and only partial similarity with more distant species like D. ananassae (melanogaster group) and D. pseudoobscura (obscura group). BS1 is located in a highly conserved region and its sequence is identical across the melanogaster group, whereas BS2 is located in a region of low conservation and not found in any of the related species. Significant similarities on slou coding and cis regulatory regions were only found between Drosophila melanogaster and the closer drosophilid species D. simulans, D. yakuba, D. erecta and D. ananassae. No homology was found using the BLAST tool (http://blast.ncbi.nlm.nih.gov/Blast.cgi) with Anopheles gambie, Apis mellifera, Xenopus tropicalis, Danio rerio, Mus musculus and Homo sapiens. (TIF) [file pgen.1002186.s004.tif]

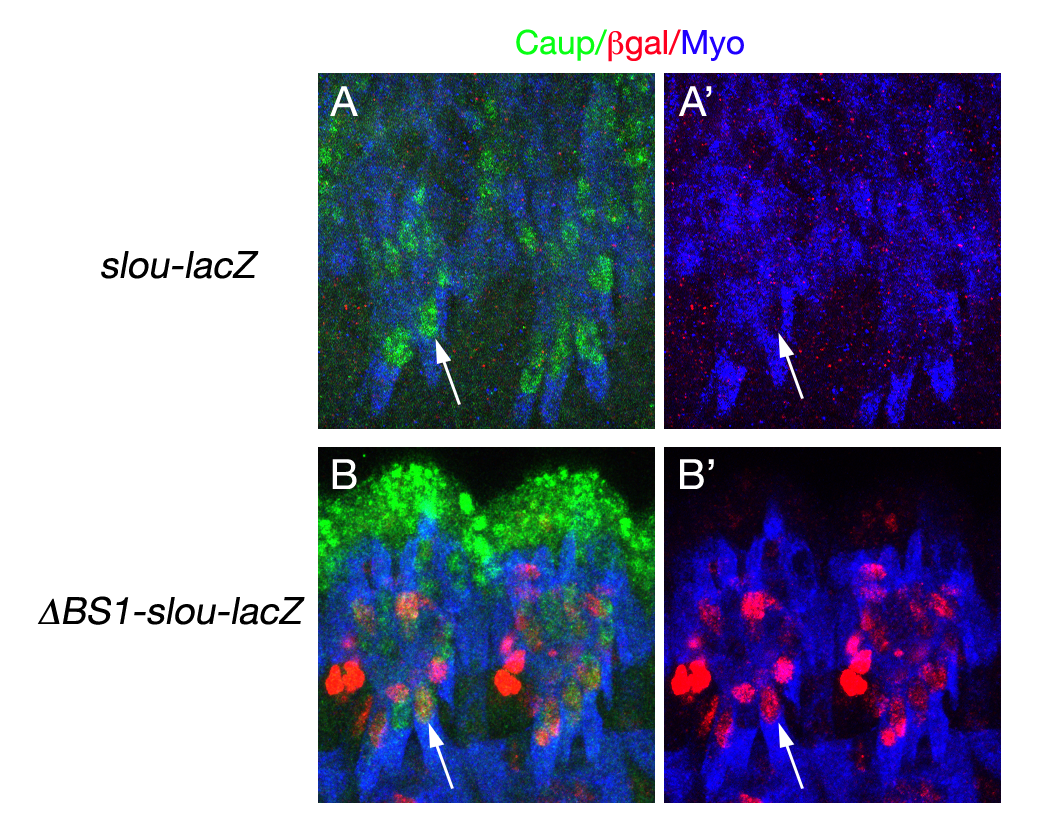

Supplement: Figure S4 — Deletion of Caup BS1 promotes lac-Z expression in LT muscles driven by slou cis-regulatory region. Lateral views of stage 15 slou-lacZ (A, A′) and ΔBS1-slou-lacZ (B, B′) embryos stained with anti-Caup (green), anti-ßgal (red) and anti-Myo (blue) antibodies. Note absence of lacZ expression in LT muscles of slou-lacZ embryos (arrows in A, A′) and co-expression of caup and lacZ in LT muscles of ΔBS1-slou-lacZ embryos (arrows in B, B′). (TIF) [file pgen.1002186.s005.tif]

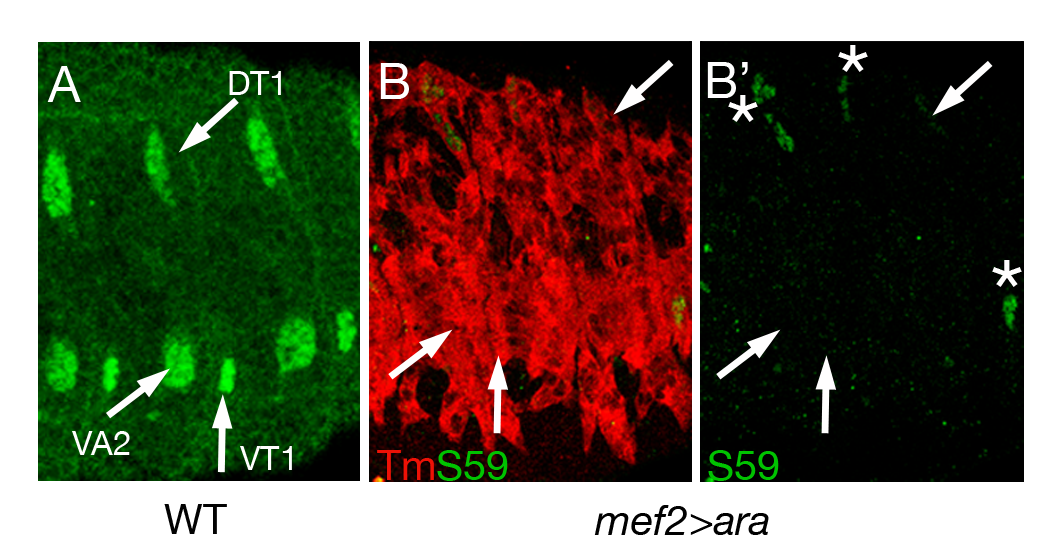

Supplement: Figure S5 — Repression of slou by ectopic expression of Ara. Lateral views of stage 15 wild-type (A) and mef2-GAL4::UAS-ara (B-B′) embryos stained with anti- Tropomyosin (red) and anti-slou (green) antibodies. (A) Note slou expression in DT1, VA2 and VT1 muscles (arrows). (B) Early expression of ara with the panmesodermal driver mef2-GAL4 represses slou in DT1, VA2 and VT1 in many segments (arrows). A few muscles maintain slou expression (asterisks). (TIF) [file pgen.1002186.s006.tif]
